# O Uso do Twitter (X) por Cardiologistas Brasileiros: Um Chamado à Ação

**DOI:** 10.36660/abc.20240711

**Published:** 2024-12-20

**Authors:** Sergio Emanuel Kaiser

**Affiliations:** 1 Universidade do Estado do Rio de Janeiro Rio de Janeiro RJ Brasil Universidade do Estado do Rio de Janeiro (UERJ), Rio de Janeiro, RJ – Brasil

**Keywords:** Mídias Sociais, Brasil, Cardiologistas

O canal de mídia proeminentemente influente, agora conhecido como X (anteriormente "Twitter") foi originalmente concebido como uma plataforma de microblog destinada a transmitir atualizações de status curtas, passíveis de entrega imediata a amigos e seguidores. No entanto, ele desencadeou uma revolução no modo de expressar e compartilhar opiniões, ao permitir o estabelecimento de conexões em tempo real. Com seu formato de mensagem curta, a plataforma permite a comunicação instantânea entre indivíduos a milhares de quilômetros de distância com apenas alguns toques na tela de um smartphone.

No contexto de algoritmos orientados por aprendizado de máquina, textos e imagens altamente emocionais impulsionam o engajamento, se espalham rapidamente e amplificam a visibilidade de anúncios incorporados ou mensagens patrocinadas. As expectativas de lucro para os proprietários da plataforma, portanto, dependem da eficiência desses algoritmos para gerar engajamento, independentemente da precisão e autenticidade das informações compartilhadas. Em 2023, os anúncios patrocinados (e personalizados) representaram 74% da receita da plataforma.^1^

Em termos de números de usuários, o X está longe de ser um dos canais de mídia online mais populares, mas seu desempenho como caixa de ressonância é extraordinário. Em abril de 2024, o X contava mais de 600 milhões de usuários ativos globalmente, colocando-o em uma modesta 12ª posição. Para efeito de comparação, o Facebook teve mais de 3 bilhões de usuários ativos durante o mesmo período.^2^ De acordo com a mesma fonte, o Brasil foi classificado como a quarta maior base de usuários, com cerca de 24,3 milhões de perfis em abril de 2024. No entanto, apesar desse ativo notável e vocal, a presença da mídia social entre a comunidade acadêmica brasileira permanece surpreendentemente limitada.^3,4^

Um número significativo de periódicos médicos, instituições e profissionais renomados mantém contas ativas no X, trocando rotineiramente conteúdo e mensagens com colegas em todo o mundo.^5^ Esse engajamento tem fomentado a cooperação entre usuários do X e publicações em periódicos médicos de alto impacto.^6^ A presença de pesquisadores no X amplia a visibilidade de seus interesses e descobertas de pesquisa, facilita a localização de pares e discussões relevantes e melhora a interação entre vários participantes.^3^ Hashtags populares, como #CardioTwitter ou #RadialFirst, focam em áreas específicas de interesse dentro de especialidades particulares e facilitam sua disseminação.

Dada a relevância de X entre a comunidade médica, o estudo "O Twitter (X) como Ferramenta de Comunicação e Educação para Cardiologistas Brasileiros: Perfil, Influência e Desafios"^7^ fornece uma análise oportuna da participação e do impacto social de cardiologistas brasileiros nesta plataforma. Através de uma ferramenta baseada na web, os autores identificaram perfis autodescritos de "cardiologistas" e filtraram profissionais brasileiros de acordo com critérios previamente especificados. Esta ferramenta também permitiu a construção de um "score de autoridade social", definido aproximadamente como o grau de influência no X gerado por um usuário individual.

Entre os principais achados deste estudo descritivo, destacou-se inicialmente uma disparidade de gênero, com grande predominância de perfis masculinos, em paralelo aos dados globais, abrindo espaço para estudos dedicados que visem melhor compreender as causas subjacentes. Não obstante, há uma presença significativa de cardiologistas do sexo feminino internacionais com alta autoridade científica sobre X. Qualquer usuário do #CardioTwitter pode encontrar vários desses perfis influentes com apenas um toque no aplicativo X de um celular.

O estudo destacou uma baixa autoridade social da maioria dos perfis estudados. É importante enfatizar que o conceito de autoridade social não reflete necessariamente a autoridade acadêmica do perfil analisado, pois métricas como nível de engajamento e contagem de seguidores influenciam esse cálculo. Alta autoridade social pode pertencer a um negacionista de estatinas altamente ativo em X, enquanto pesquisadores proeminentes com influência acadêmica significativa podem nem mesmo ter contas nesta plataforma. Outro fator a ser considerado é a natureza dinâmica desta métrica. Os dados neste estudo foram coletados em um ponto de tempo específico, vários meses antes de sua publicação. Uma revisão atual dos vinte perfis listados na Tabela 3 revela pelo menos seis que não devem mais ser pontuados para autoridade social. Por exemplo, o perfil "flaviobessajr" não existe mais. Dois perfis listados – "carlosF201634" e "AdrianaSerpa1" – agora são privados, o que atualmente representaria um critério de exclusão para o estudo; possivelmente, esses perfis eram públicos no momento da extração de dados.

Não obstante as questões acima mencionadas, destaca-se o baixo engajamento e a relevância limitada dos cardiologistas brasileiros no X. A barreira linguística apontada pelos autores é provavelmente a principal explicação para esse achado. Outra possível explicação merece a atenção das agências brasileiras de fomento à pesquisa: em 2022, a produção científica brasileira caiu 7,2% em relação aos anos anteriores, contrastando fortemente com o aumento progressivo observado em anos anteriores.^8^ Segundo a mesma fonte, a contribuição do Brasil para as publicações científicas globais entre 2018 e 2022 nunca ultrapassou 3%. À luz desses números, este estudo serve como um chamado à ação para os profissionais de saúde engajados em atividades acadêmicas. Na própria experiência do autor deste editorial, o X representa uma oportunidade valiosa para a colaboração científica entre pesquisadores brasileiros e internacionais. É, portanto, digna de elogio a contribuição dos autores deste estudo, cujo compartilhamento será bem vindo no @arquivosSBC, o perfil no X do periódico ABC Cardiol.
