# Supplementary material for: O Uso do Twitter (X) por Cardiologistas Brasileiros: Um Chamado à Ação
Source: Arq Bras Cardiol. 2024 Dec 20;121(12):e20240711. [Article in Portuguese] doi: 10.36660/abc.20240711 (PMC11809857; doi:10.36660/abc.20240711)
Supplement: Translation

# Translation: The Use of Twitter (X) by Brazilian Cardiologists: A Call to Action

**DOI:** 10.36660/abc.20240711i

**Authors:** Sergio Emanuel Kaiser

**Affiliations:** 1 Rio de Janeiro RJ Brazil Universidade do Estado do Rio de Janeiro (UERJ), Rio de Janeiro, RJ – Brazil

**Keywords:** Social Media, Brazil, Cardiologists

The prominently influential media outlet currently known as X (Formerly "Twitter") was originally conceived as a microblogging platform intended to broadcast short status updates, amenable to immediate delivery to friends and followers. However, it has sparked a revolution in the way information is shared, opinions are expressed, and connections that are made in real-time. With its short-message format, the platform enables instant communication between individuals thousands of miles apart with just a few taps on a smartphone screen.

In the framework of machine-learning driven algorithms, highly emotional texts and images drive engagement, spread rapidly, and amplify visibility for embedded ads or sponsored messages. Profit expectations for the platform's owners, therefore, rely on the efficiency of these algorithms to generate engagement, regardless of the accuracy and authenticity of the shared information. By 2023, sponsored (and tailored) ads accounted for 74% of the platform's revenue.^1^

In terms of user numbers, X is far from being one of the most popular online media channels, but its performance as a sounding board is extraordinary. In April 2024, X counted over 600 million active users globally, placing it in a modest 12th position. For comparison, Facebook had over 3 billion active users during the same period.^2^ According to the same source, Brazil ranked as the fourth-largest user base, with about 24.3 million profiles in April 2024. However, despite this notable and vocal asset, the presence of social media among the brazilian academic community remains surprisingly limited.^3,4^

A significant number of medical journals, institutions, and renowned professionals maintain active accounts on X, routinely exchanging content and messages with colleagues worldwide.^5^ This engagement has fostered cooperation among X users and publications in high-impact medical journals.^6^ The presence of researchers on X broadens the visibility of their interests and research findings, facilitates locating peers and relevant discussions, and enhances interaction among various participants^3^. Popular hashtags, such as #CardioTwitter or #RadialFirst, focus on specific areas of interest within particular specialties and facilitate their dissemination.

Given X's relevance among the medical community, the study "Twitter (X) as a Communication and Education Tool for Brazilian Cardiologists: Profile, Influence and Challenges"^7^ provides a timely analysis of the participation and social impact of Brazilian cardiologists on this platform. Using a web-based tool, the authors identified self-described "cardiologist" profiles and filtered brazilian professionals according to previously specified criteria. This tool also enabled the construction of a "social authority score," roughly defined as the degree of influence on X generated by an individual user.

Among the primary findings of this descriptive study, a gender disparity initially stood out, with a large predominance of male profiles, in parallel with global data, leaving room for dedicated studies aimed at better understanding the underlying causes. Nontheless, there is a significant presence of international female cardiologists with high scientific authority on X. Any #CardioTwitter user can find several of these influential profiles with just a tap on a mobile phone X app.

The study highlighted a low social authority of most profiles studied. It is important to emphasize that the concept of social authority does not necessarily reflect the academic authority of the analyzed profile, as metrics like engagement level and follower count factor into this calculation. High social authority could belong to a statin denialist highly active on X, while prominent researchers with significant academic influence may not even have accounts on this platform. Another factor to consider is the dynamic nature of this metric. The data in this study were collected at a specific time point, several months before its publication. A current review of the twenty profiles listed in Table 03 reveals at least six that should no longer be scored for social authority. For example, the profile "flaviobessajr" no longer exists. Two profiles listed – "carlosF201634" and "AdrianaSerpa1" – are now private, which would currently represent an exclusion criterion for the study; possibly, these profiles were public by the time of data extraction.

Notwithstanding the aforementioned issues, a low engagement and limited relevance of brazilian cardiologists on X stand out. The language barrier noted by the authors is likely the primary explanation for this finding. Another possible explanation deserves the attention of brazilian research funding agencies: in 2022, brazilian scientific production dropped by 7.2% compared to previous years, contrasting sharply with the progressive increase seen in earlier years.^8^ According to the same source, Brazil's contribution to global scientific publications between 2018 and 2022 never exceeded 3%. In light of these figures, this study serves as a call to action for health professionals engaged in academic activities. In the very experience of the author of this editorial, X represents a valuable opportunity for scientific collaboration between brazilian and international researchers. The authors of this study are to be praised for their contribution, which should be expected to be shared on @ArquivosSBC, the journal profile of ABC Cardiol on X.
